# Syntaxin 16’s Newly Deciphered Roles in Autophagy

**DOI:** 10.3390/cells8121655

**Published:** 2019-12-17

**Authors:** Bor Luen Tang

**Affiliations:** 1 Department of Biochemistry, Yong Loo Lin School of Medicine, National University of Singapore, Singapore 117596, Singapore; bchtbl@nus.edu.sg; Tel.: +65-6516-1040; 2 NUS Graduate School for Integrative Sciences and Engineering, National University of Singapore, Singapore 119077, Singapore

**Keywords:** ATG9, autophagy, autophagosome, SNARE, syntaxin 16, syntaxin 17, VAMP7

## Abstract

Syntaxin 16, a Qa-SNARE (soluble *N*-ethylmaleimide-sensitive factor activating protein receptor), is involved in a number of membrane-trafficking activities, particularly transport processes at the trans-Golgi network (TGN). Recent works have now implicated syntaxin 16 in the autophagy process. In fact, syntaxin 16 appears to have dual roles, firstly in facilitating the transport of ATG9a-containing vesicles to growing autophagosomes, and secondly in autolysosome formation. The former involves a putative SNARE complex between syntaxin 16, VAMP7 and SNAP-47. The latter occurs via syntaxin 16’s recruitment by Atg8/LC3/GABARAP family proteins to autophagosomes and endo-lysosomes, where syntaxin 16 may act in a manner that bears functional redundancy with the canonical autophagosome Qa-SNARE syntaxin 17. Here, I discuss these recent findings and speculate on the mechanistic aspects of syntaxin 16’s newly found role in autophagy.

## 1. Introduction

Vesicular membrane trafficking [1] and macroautophagy [2] are two highly conserved cellular membrane remodeling processes in eukaryotes. The former mediates the transfer of materials between membrane compartments, while the latter serves to degrade and recycle cytosolic as well as membranous cellular materials. During vesicular membrane trafficking in the exocytic and endocytic pathways, vesicular intermediates are generated from donor membranes via membrane-curving and cargo-sequestering coat protein complexes [3], and these eventually fuse to target membranes with the aid of soluble *N*-ethylmaleimide-sensitive factor activating protein receptors (SNAREs) [4]. On the other hand, autophagy begins by autophagosome formation or biogenesis, ending with the latter’s eventual fusion with the lysosome [5]. Autophagosome biogenesis is dependent on the action of several protein complexes, encoded by a large conserved set of autophagy (Atg) related genes [6] that have no apparent cross-activity in vesicular membrane traffic. However, autophagosome biogenesis is known to be aided by different coat protein complexes, and the process of autophagosome-lysosome fusion is obligatorily dependent on a number of SNAREs with known functions in vesicular membrane traffic.

Multiple subcellular sites could give rise to nascent autophagosomes. Autophagosome biogenesis has been shown to occur at locations as varied as endoplasmic reticulum (ER)-mitochondria contact sites [7], ER exit sites [8,9], the trans-Golgi network (TGN) [10], ER-plasma membrane contact sites [11] and the recycling endosomes [12]. The diversity of autophagosomal origin is reflected by the fact that most of the known coat protein complexes in the exocytic and endocytic pathways, including coat protein I (COPI) [13], COPII [14,15,16,17], AP1 [10], AP2 [18] and AP4 [19,20], have all been implicated in the supply of membrane sources for autophagosome formation. In this regard, perhaps the most prominent are ER-derived COPII vesicles [14,15,16,17,21,22,23,24] and Atg9-containing, endosome-derived vesicles [18,19,20,25,26,27,28,29]. The multiple membrane-spanning Atg9 could be found at membranes of the TGN, recycling endosomes as well as a subpopulation of cytoplasmic membrane vesicles, and upon autophagy induction translocates to sites of autophagosome biogenesis [30,31]. This vesicle-targeting process is also known to involve certain SNARE molecules [28,32] and tethering complexes [33,34].

Autophagy is typically induced upon cellular stress and nutrient deprivation, which is sensed by signaling complexes [35] such as the mechanistic target of rapamycin (mTOR) complex 1 (mTORC1) [36] and liver kinase B1 (LKB1)–AMP-activated protein kinase (AMPK) [37]. During the initiation of autophagosome biogenesis, the Atg1/Unc51-like kinase 1 (ULK1)-containing complex activates a second complex containing Atg6/Beclin1 and the phosphatidylinositol 3-kinase Vps34 [38]. The production of phosphatidylinositol (3,4,5)-trisphosphate (PIP3) provides a molecular docking platform for recruitment of more factors and the formation of a pre-autophagosome structure, termed the phagophore [39]. Expansion of phagophore encloses cytoplasmic contents, and its subsequent membrane closure and fission generates the double-membraned autophagosome.

A key process herein is the phosphoethanolamine (PE)-lipidation [40] of members of the Atg8/microtubule-associated proteins 1A/1B light chain 3 (LC3)/ gamma-aminobutyric acid receptor-associated protein (GABARAP) family [41] by two ubiquitin-like conjugation systems [42]. Functioning as an E2-like enzyme, Atg3 forms an active intermediate with Atg8/LC3/GABARAP proteins that is recruited to the lipidation site. LC3 is first processed at its C terminus by the cysteine peptidase Atg4 to LC3-I, with the latter subsequently conjugated with PE to LC3-II by the E3-like Atg16/Atg16L–Atg5 complex [43]. In this regard, LC3 and its lipidated form LC3-II decorates the autophagosome, and is the most widely used autophagy marker [44]. The Atg8/LC3/GABARAP proteins are essential for autophagosome biogenesis [45], and appear to function in selective capture of autophagic cargo [46], acting analogously to the ubiquitin-binding autophagy receptors such as p62/ sequestosome-1 (SQSTM1) [47]. On the other hand, the Atg8/LC3/GABARAP proteins have also been shown to be crucial for autophagosome-lysosome fusion, but not autophagosome formation [48].

Fusion of autophagosome and lysosome to form the autolysosome requires specific SNAREs [49]. The best known SNARE in this regard is the Qa-SNARE syntaxin 17 (STX17) [50,51]. Together with its cognate SNARE partners, the Qbc SNARE Snap29 and either one of the R-SNAREs VAMP7 or 8, the STX17-containing trans SNARE complex mediates autolysosome formation [52,53]. STX17 also interacts with membrane tethers, including Atg14/Atg14L [54], as well as the homotypic fusion and vacuole protein sorting (HOPS) complex previously known for its role in the endocytic pathway [55,56], thus modulating a docking step prior to fusion. STX17 is apparently targeted to the outer autophagosomal membrane by a unique C-terminal hairpin structure consisting of tandem transmembrane domains with glycine zipper-like motifs [52]. Its autophagosomal recruitment is also mediated by interactions with immunity-related GTPase family M protein (IGRM) and LC3 [57]. Interestingly, STX17 has also been implicated in phagophore closure [7] and in regulating autophagy initiation [58]. The longin SNARE Ykt6 has also been recently implicated in autophagosome-lysosome fusion, although its mode of action remains to be precisely determined [59,60,61,62].

Several recent advances have now furthered our understanding of the role of another SNARE, syntaxin 16 (STX16) in autophagy. STX16 is a TGN-localized Qa-SNARE [63,64] known mainly for its role in endosomal-Golgi retrograde transport [65,66]. In an earlier study from Klionsky’s group, the yeast SNARE Tlg2 has been implicated in Atg9 transport and thus a potential role in autophagosome biogenesis [32]. However, a role for STX16, the mammalian homologue of Tlg2 [67], in autophagy has been unclear. Interestingly, recent findings now indicate that STX16 has a dual role, both in autophagosome formation [28], as well as in autolysosome biogenesis [68]. In the paragraphs below, these findings are discussed together with some mechanistic speculations on STX16’s dual roles in autophagy.

## 2. Syntaxin 16’s Involvement in Autophagosome Formation

In an earlier reported work, Aoyagi and colleagues studied the role of the R-SNARE VAMP7 in metabolic dysfunction [69]. VAMP7 is upregulated in both wild-type mice fed a high-fat diet and in diabetic leptin receptor mutant db/db mice, while pancreatic β-cell-specific knockout of VAMP7 disrupted glucose-stimulated ATP production and insulin secretion. VAMP7-deficient β-cells have defective autophagosome formation and impaired mitochondrial functions, with apparent mitochondrial accumulation of p62/SQSTM1. These pathological phenotypes of VAMP7-deficient β-cells are worsened in mice fed with a high-fat diet. Autophagy impairment resulting from VAMP7 deficiency could thus underlie the accumulation of dysfunctional mitochondria, resulting in consequential impairment in β-cell function.

How exactly does a loss of VAMP7 impair autophagy in pancreatic β-cells? This question is now addressed in a follow up study by Aoyagi and colleagues [28]. The authors noted that VAMP7 resides on Atg9a-containing vesicular fractions from Rab11-positive recycling endosomes (REs) in a β-cell derived Min6 cell line. Although co-immunoprecipitation of HA-tagged VAMP7 expressed in VAMP7-deficient cells and Atg9a could not be demonstrated in lysates with detergent, the amount of ATG9a recovered in HA-VAMP7 enriched vesicles is markedly increased by autophagy induction. Furthermore, imaging analyses showed that only the punctated signals of autophagy proteins acting downstream of ATG9a (such as WD-repeat protein interacting with phosphoinositides (WIPI2) and ATG14L), but not those acting upstream, are diminished in VAMP7-deficient cells. VAMP7-deficiency thus appears to disrupt autophagosome formation that occurs via the route of, or otherwise fueled by, ATG9a vesicles. VAMP7 is a R-SNARE with an N-terminal longin domain [70], and the latter mediates the SNARE molecule’s interaction with a group of regulatory proteins. One of these, the clathrin adaptor and ARFGAP HIV-1 Rev-binding protein (HRB) [71], appears to be involved in VAMP7’s role in autophagy, as its silencing reduced the number of LC3-II puncta. Interestingly, silencing of HRB also resulted in a shift of the colocalized signals of VAMP7 and ATG9a to the plasma membrane instead of the REs, as demonstrated by total internal reflection fluorescence (TIRF) microscopy. The VAMP7-interacting HRB may thus act to shift VAMP7 and ATG9a from the plasma membrane to REs, where the latter might decorate vesicles en route to an autophagosome formation site.

As a SNARE molecule, VAMP7 would presumably function in the context of a SNARE complex. The authors identified by co-immunoprecipitation and peptide sequencing two robust SNARE interacting partners for VAMP7, namely the Q-SNAREs STX16 and SNAP-47. These two SNAREs showed partial colocalization with VAMP7 and ATG9a in Min6 cells, and silencing of either STX16 or SNAP-47 reduced LC3-II puncta. Of further pathophysiological relevance is the finding that silencing of either HRB, STX16 or SNAP-47 all resulted in some degree of mitochondrial dysfunction and impairment in glucose-stimulated insulin secretion. Taken together, the findings of Aoyagi et al. [28] indicate that the SNAREs VAMP7, STX16 and SNAP-47 likely form a functional SNARE complex involved in mediating autophagosome formation in a route where RE-derived ATG9a-containing vesicles serve as a membrane source. This is reminiscent of, albeit in a limited way, the earlier finding with STX16’s homolog, Tlg2, which was shown to be important for Atg9 trafficking in yeast [32].

## 3. Syntaxin 16’s Involvement in Autolysosome Biogenesis

As it turns out, autophagosome biogenesis is not the only role STX16 has in autophagy, as recent work has also implicated it in a somewhat unexpected role of autolysosome formation [68]. On the basis of their earlier finding that STX17 harbors a LC3-interacting region (LIR) [72] for mammalian Atg8/LC3/GABARAP family members, and is thereby recruited onto autophagosomes, Deretic’s group screened and found more SNAREs with LIRs [68]. Interestingly, the only R-SNARE identified in this regard is VAMP7 [68]. Several Q-SNAREs with a wide range of cellular membrane distributions have LIRs, including ER-localized STX18 [73], plasma membrane-enriched STX3 and STX4 [74], as well as the Q-SNARE members of a SNARE complex at the TGN, STX6, STX16 and VTI1 [65]. Focusing on STX16, the authors defined a critical motif of its LIR as the 4-amino acid sequence of ^219^LVLV^222^, and showed that this motif interacts with GABARAP’s LIR docking site.

Interestingly, neither STX17 nor STX16 knockout in human cell lines such as HeLa and Huh7 using the CRISPR/Cas9 technique has a significant effect on autophagic flux. The phenotype due to the loss of STX17 is somewhat milder than some of those previously reported in other contexts [52,53,62]. However, when both SNAREs were ablated together, there was a complete abrogation of LC3 flux, with LC3-II accumulating to a level comparable to the condition when cells are treated with the lysosomal pH-disrupting vacuolar type H^+^-ATPase inhibitor bafilomycin A1. This phenotype could be partially reversed by exogeneous over-expression of STX16. Autolysosome formation thus appears to be completely blocked in cells deficient in both STX16 and STX17. The STX16–STX17 double-knockout cells also inhibited specific autophagic processes including mitophagy, pexophagy, xenophagy and ribophagy to varying degrees. These observations suggest that STX16 could potentially replace the function and activity of STX17 in autophagy.

STX16 is previously known to function in endosome-TGN retrograde membrane traffic [65,66], as well as GLUT4 trafficking to the cell surface [75]. It also has a number of other known functions ranging from neurite extension to cytokinesis [76,77,78,79]. In the context of the autophagy pathway, what role(s) does STX16 play exactly? The authors found that STX16-knockout cells have reduced signals and diminished levels of the lysosomal membrane proteins LAMP1 and LAMP2 under both basal and autophagy-inducing starvation conditions. Furthermore, the component SNAREs of the STX16-containing complex, including STX6 and STX16, interacts with the HOPS complex [80,81] component VPS41 but not VPS33. As VPS41 (together with VAMP7) was specifically implicated in the TGN-late endosome transport of lysosomal membrane proteins [82] such as LAMP1 and LAMP2, STX16 appears to play a role in lysosome biogenesis. Furthermore, tracking of acidified compartments with Lysotracker in STX16 knockout cells under autophagy-inducing conditions showed that redistribution of Lysotracker-positive structures changes from largely cytoplasmic to being clustered around a TGN46-marked perinuclear region. This STX16 knockout phenotype could however be contributed to by starvation-induced perinuclear repositioning of lysosomes [83]. In line with the above observations, interaction between STX16 and the lysosomal R-SNARE, VAMP8, increased significantly under starvation conditions.

These STX16-knockout associated changes in the lysosomal compartments has direct consequences on mTOR localization and signaling. Active mTOR is localized to lysosomal membranes [84], and upon starvation-induced autophagy becomes cytoplasmic. STX16-knockout reduced active mTOR signaling, as assessed by phosphorylation of the key mTOR kinase substrates 4E-BP1 and ULK1, and enhanced its cytoplasmic translocation upon autophagy induction. Combinatory knockout of mammalian Atg8s in HeLa cells affected the co-localization of STX16 and LAMP2, reduced interactions between components of the STX16/STX6/VTI1a SNARE complex, as well as mTOR inactivation in response to starvation. STX16’s autophagy-related function in the endolysosomal system is thus dependent on its recruitment to these membranes by members of the mammalian Atg8/LC3/GABARAP family.

## 4. New Perspectives

**Figure 1:**
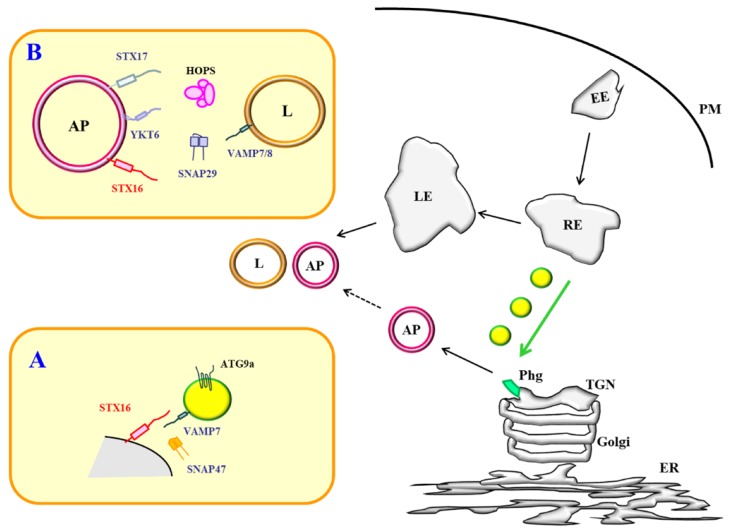
A schematic diagram depicting the two modes of STX16 action in autophagy. (**A**). Syntaxin 16 (STX16) could function at a trans-Golgi network (TGN)-endosomal phagophore formation site to facilitate trafficking of ATG9a-containing vesicles from the recycling endosome, in conjunction with SNARE partners VAMP7 and SNAP-47. (**B**) STX16 could function in autophagosome–lysosome fusion, potentially replacing STX17. PM—plasma membrane; EE—early endosome; RE—recycling endosome; Phg—phagophore; TGN—trans-Golgi network; Golgi—Golgi apparatus; ER—endoplasmic reticulum; AP—autophagosome; L—lysosome.

The new findings outlined above broadens the cellular roles of the SNAREs STX16 and VAMP7. In particular, STX16 is now more firmly established as a key player in autophagy in addition to its previously known roles in membrane trafficking. VAMP7’s potential role in autophagosome-lysosome fusion has been previously reported [53,60,85]. It now appears that it also has a role in autophagosome biogenesis. Intriguingly therefore, both an R-SNARE and a Q-SNARE are now known to have dual functions in the autophagy process. STX16 and VAMP7, at least in mammalian cells, shares two features. The first, as highlighted by the findings of Aoyagi et al. [28], is that they could form a SNARE complex in pancreatic β-cells to facilitate delivery of Atg9a containing, RE-derived vesicles to a forming autophagosome, likely at a membrane location near the TGN. In this regard, both are important for autophagosome biogenesis, at least via the post-Golgi route involving ATG9a bearing membranes (see Figure 1).

Secondly, both VAMP7 and STX16 harbor LIRs and could potentially be recruited by the Atg8/LC3/GABARAP proteins to autophagosomes [68]. In this regard, both could be viewed to be facilitating autophagosome ’maturation’, in the sense of the latter acquiring lysosomal fusion competency with time, as per its recruitment of STX17 [86]. However, while VAMP7 has been previously demonstrated to function in the autophagosome–lysosome fusion SNARE complex involving STX17 [53,60,85], likely in an interchangeable manner with VAMP8, the exact mode of action for STX16 in this latter regard is still unclear. A potential caveat, arising when the studies above are considered together, is that manipulations such as depletion of STX16 (as well as VAMP7) may hit the autophagy pathway in more than one place, which could complicate interpretations.

What exactly does STX16 do in terms of autophagosome–lysosome fusion or autolysosome formation? From the STX16 and STX17 single- and double-knockouts, it would appear that STX16 and STX17 share redundant functions in autophagosome–lysosome fusion, as loss of either has no significant phenotype while ablation of both almost completely abolished autophagic flux. In support of this notion, STX16, like STX17, could be recruited via its LIR to autophagosomes [68]. STX16 is a Qa-SNARE, and could therefore directly replace STX17 in fulfilling the stoichiometric requirement for a functional, fusion-mediating SNARE complex [87]. Based on the limited data of tagged STX16 capable of capturing the HOPS component VPS41 [68], STX16, like STX17, could engage tethering capacity for autophagosomes. Other recent studies have implicated another Longin R-SNARE, YKT6, in autophagosome–lysosome fusion. Results from two different experimental models, however, allowed YKT6’s mode of action to be interpreted differently. In HeLa cells, YKT6 could function as an autophagosomal R-SNARE to engage a lysosomal Q-SNARE, and mediate fusion independently of STX17 [62]. The latter accounts for residual autophagic flux in STX17 knockout cells. However, in *Drosophila* larvae fat cells, Ykt6 acts as a lysosomal R-SNARE, forming a complex with Stx17 and Snap29. This complex is, however, less stable than the canonical Vamp7-Stx17-Snap29 complex, and Vamp7 is readily displaced Ykt6 [60]. How does STX16 fit into the scheme of things in autolysosome formation is unclear at the moment. A straightforward mode of action would be that having been recruited by Atg8/LC3/GABARAP, STX16 acts as an autophagosomal Q-SNARE like STX17 and forms a fusion competent SNARE complex with SNAP29 and VAMP7/8. The existence of such a SNARE complex and its functionality has, however, not yet been demonstrated. Alternatively, and particularly in view of its recruitment and activities at the endo-lysosomal membrane as demonstrated by Gu et al. [68], STX16 could potentially also act as a lysosomal Q-SNARE in mediating fusion.

An important question that remains unanswered pertains to the notion of the autophagy process being very much cell- and context-dependent. This is clearly the case for autophagosome biogenesis, which could be initiated from multiple subcellular sites and be fueled by different membrane sources. It is increasingly likely that the mode and mechanism of autophagosome-lysosome fusion could likewise be fairly varied. In other words, which SNAREs are used or engaged in a particular fusion event differ between cell types and physiological contexts. A related possibility is that even the membrane origin of an autophagosome would dictate its SNARE profile. It is conceivable that given STX17’s more pre-Golgi/ER localization [88], it would be more readily recruited by autophagosomes forming via ER-mitochondrial contact sites or ER exit sites [8,9] that are dependent on COPII-mediated vesicular traffic [14,15,16,17,24] for membrane sources. On the other hand, autophagosomes formed at a more TGN-endosomal site may acquire STX16 more easily due simply to the apparent proximity of the STX16-bearing membrane compartments.

From another perspective, having a Q-SNARE (STX16) and an R-SNARE (VAMP7) at both the beginning and the end point of the autophagy process could be a way to coordinately regulate autophagosome formation and its eventual lysosomal fusion. STX17 has likewise been shown to regulate initiation of autophagy through its phosphorylation by TANK-binding kinase 1 (TBK1) [58]. Such coordinated or multivalent regulation of autophagy has also been demonstrated for the small GTPase RAB2, which was recently shown to regulate both autophagosome and autolysosome formation [89]. Based on its primary sequence, STX17 is an ‘ancient’ SNARE [90] and the most divergent amongst the syntaxin family of proteins, which is somewhat in line with its rather specialized role in autolysosome formation. Conversely, STX16 has multiple known roles in membrane trafficking [78]. In ways that remains to be deciphered, it is therefore conceivable that STX16’s availability and recruitment to different membrane could serve as a regulatory link between vesicular membrane traffic and autophagy at the TGN-endosomes. These possibilities remain to be explored.
